# Morphological Structures and Self-Cleaning Properties of Nano-TiO_2_ Coated Cotton Yarn at Different Washing Cycles

**DOI:** 10.3390/nano13010031

**Published:** 2022-12-21

**Authors:** Mirra Edreena Sallehudin, Nor Dalila Nor Affandi, Ahmad Mukifza Harun, Mohammad Khursheed Alam, Liliana Indrie

**Affiliations:** 1 Textile Research Group, Faculty of Applied Sciences, Universiti Teknologi MARA, Shah Alam 40450, Selangor, Malaysia; 2 Nano Lab, Faculty Engineering, University Malaysia Sabah, Kota Kinabalu 88400, Sabah, Malaysia; 3 College of Dentistry, Jouf University, Sakaka 72721, Saudi Arabia; 4 Department of Dental Research Cell, Saveetha Dental College and Hospitals, Saveetha Institute of Medical and Technical Sciences, Chennai 72345, India; 5 Department of Public Health, Faculty of Allied Health Sciences, Daffodil International University, Dhaka 1216, Bangladesh; 6 Department of Textiles, Leather and Industrial Management, Faculty of Energy Engineering and Industrial Management, University of Oradea, Universitatii Str. No. 1, 410087 Oradea, Romania

**Keywords:** morphology, self-cleaning, titanium dioxide, cotton, washing cycles

## Abstract

Titanium dioxide (TiO_2_) has an extraordinary photocatalytic activity and it effectively provides self-cleaning properties for cotton products. With the presence of succinic acid, it helps the adherence of the TiO_2_ nanoparticles on cotton surfaces. However, the ability of succinic acid to keep the TiO_2_ adhered on cotton after washing is not yet fully understood. Therefore, this study aimed to investigate the effects of washing cycles on nano-TiO_2_ coated cotton yarn with the aid of succinic acid on the morphological structures and self-cleaning properties. In this study, the nano-TiO_2_ was synthesized using a hydrothermal method. The cotton yarn was coated with succinic acid and was later dipped in a nano-TiO_2_ nanoparticles suspension. The nano-TiO_2_ coated yarn samples then underwent the 5^th^, 10^th^, 15^th^, and 20^th^ wash cycles and were tested for morphological structures and self-cleaning. The self-cleaning properties of the nano-TiO_2_ coated yarn were determined using the depth of colour stain. The depth of the colour stain was presented as K/S value, where K and S are corresponded to the absorption and scattering coefficients of the stained fabric, respectively. From the analysis, our synthesized nano-TiO_2_ had a size of 20–50 nm range with a band gap of 3.06 eV. After coating, the nano-TiO_2_ coated cotton yarn changed in its morphological structure at 5^th^, 10^th^, 15^th^, and 20^th^ wash cycles, respectively. At the 20^th^ wash cycle, the weight (%) of the Ti element continued to decrease up to 4.45%, reducing the photocatalytic activity with the K/S value close to the stained yarn, which was about 0.4. The 5^th^ wash cycle maintained a good photocatalytic activity with the K/S value of 0.06 near to the K/S value of the unstained cotton yarn. The presence of succinic acid in the nano-TiO_2_ coated cotton yarn provided good self-cleaning properties up to the 15^th^ wash cycle. By undertaking this study, an enhanced cotton property has been developed that will benefit the textile and clothing industry. This nano-TiO_2_ coated cotton the has potential to be used for daily apparel and sportwear.

## 1. Introduction

In recent years, textiles applied with titanium dioxide have shown several outstanding properties, such as it improves the antibacterial property of textile materials [1,2], anti-static [3], stain resistant [4], conductive [5], and UV protection [6,7]. In addition, titanium dioxide is greatly valued by a more demanding and discerning consumer market as high-value-added products. Titanium dioxide (TiO_2_) is a powder-based chemical and any material based on titania has a great potential for the application of self-cleaning and anti-bacterial coatings [8,9]. Sol-gel, chemical vapour deposition, electrochemical deposition and hydrothermal are methods to produce nano-TiO_2_. Hydrothermal synthesis is a common method to produce high yields of nano-TiO_2_ [10]. As reported by Liu et. al, the benefits of hydrothermal synthesis are that it is a simple method to produce nano-size morphology for a large-scale production. Several modifications can be done to enhance the attributes of titanium dioxides, and they are also high in cation-exchange capacity and length-to-diameter ratio [10]. Due to these promising features, the current study has chosen the hydrothermal method to synthesize nano-TiO_2_.

In addition, the TiO_2_ is widely studied for photocatalysis. The photoreaction activity of the TiO_2_ can be enhanced by various factors, such as crystallinity, morphology, and surface area [11]. The large band gap of titanium dioxide, which is around 3.0 to 3.2 electron volts, affects its light absorption ability [12,13]. Due to its surface area and size, it can only absorb a short wavelength of light, which falls in the UV region. According to Rambabu et al. [14], the other factors that limit the commercial applications of titanium dioxide are its spectral response and fast charge recombinant. As a result, the commercial applications of the TiO_2_ are relatively limited [14]. Therefore, there is a crucial need to improve the photocatalyst synthesizing method to produce the ideal photocatalyst and enhance its applications globally. Hence, we have modified the synthesizing method to produce nano-titania(nano-TiO_2_) particles. The nano-TiO_2_ was then coated with cotton yarn for self-cleaning properties.

Even though the TiO_2_ nanoparticles exhibit several promising properties, however, it is a challenging task, because every successful incorporation of TiO_2_ into textiles faces a low surface area of the support and weak interactions between the fibre and nanoparticles [15]. As a result, the adherence of TiO_2_ nanoparticles on the surface of fabric is usually not strong enough, and eventually comes off, especially after washing [15].

**Figure 1:**
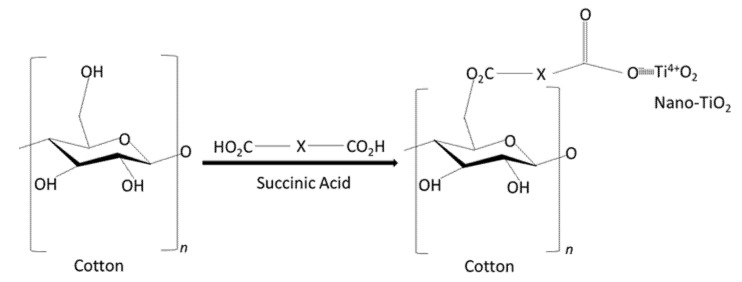
Chemical bonds between succinic acid, cellulose, and nano-TiO_2._ Figure is adapted and modified from [22].

Although there are several approaches from previous studies to improve the adherence of titanium dioxide nanoparticles on fabric surfaces, these approaches provide some weaknesses. For example, Bozzi [16] functionalized a cotton fabric surface through the activation of RF-plasma, MW-plasma, and UV-irradiation, which formed a functional group negatively charged to anchor TiO_2_ on the cotton fabric surface. The same methods were also implemented by Daoud [9]. However, this method weakens the fibre’s strength [17]. The second method is by using a padding mangle to coat titanium dioxide nanoparticles onto fabric surfaces. Kale et al. [18] coated cotton fabric with cellulose-TiO_2_ by a roller padding for self-cleaning properties. Xu et al. [19] and Yuranova et al. [20] studied the coating of TiO_2_ on cotton for photocatalytic self-cleaning and binding ability of TiO_2_ on fabric surfaces. However, these padding mangle methods affect the stiffness of the fabric. To overcome these issues, several coating agents were used to enhance the adherence of the TiO_2_ particles onto a textile material. Succinic acid was reported to provide good adhesion of the TiO_2_ on fabric [21]. Cheng and Wang [21] reported that functional groups of the succinic acid could interact with nanometer titanium dioxide, enhancing the crosslinking between the succinic acid and cellulose molecules from cotton. A previous study reported that carboxylic acids such as succinic acid were commonly used to attach TiO_2_ on cotton cellulose [22]. As illustrated in Figure 1, the succinic acid can form an ester bond between carboxylic group of dicarboxylic and hydroxyl group of cellulose polymer chains from cotton. To accelerate the ester formation reactions, sodium hypophosphite was added to the cotton samples [22]. From these previous findings, the succinic acid and sodium hypophosphite were selected and used in this current study. However, the ability of the succinic acid to maintain the physical structures of TiO_2_ coated cotton yarn and self-cleaning properties after washing is not yet fully understood. Hence, the current study aimed to investigate the effects of washing cycles of our synthesized nano-TiO_2_ coated cotton yarn with the aid of succinic acid on the morphological structures and self-cleaning properties of the yarn.

## 2. Materials and Methods

### 2.1. Materials

The nano-titania (nano-TiO_2_) particles were prepared through the hydrothermal synthesis. A description of the method can be found elsewhere [23,24]. The commercial TiO_2_ (Sigma-Aldrich, nano-powder with the particle size of 25 nm, 99.7% purity), succinic acid (analytical grade) by Sigma-Aldrich, sodium hypophosphite by Sigma-Aldrich, and 100% cotton yarn were used without further purification. The undoped TiO_2_ was synthesized using a modified hydrothermal process with the TiO_2_ purity of 92%. The synthesis process was carried in Universiti Malaysia Sabah and is described elsewhere [25,26].

### 2.2. Preparation of Nano-Titania (Nano-TiO_2_) Suspension and Coating Process

**Figure 2:**
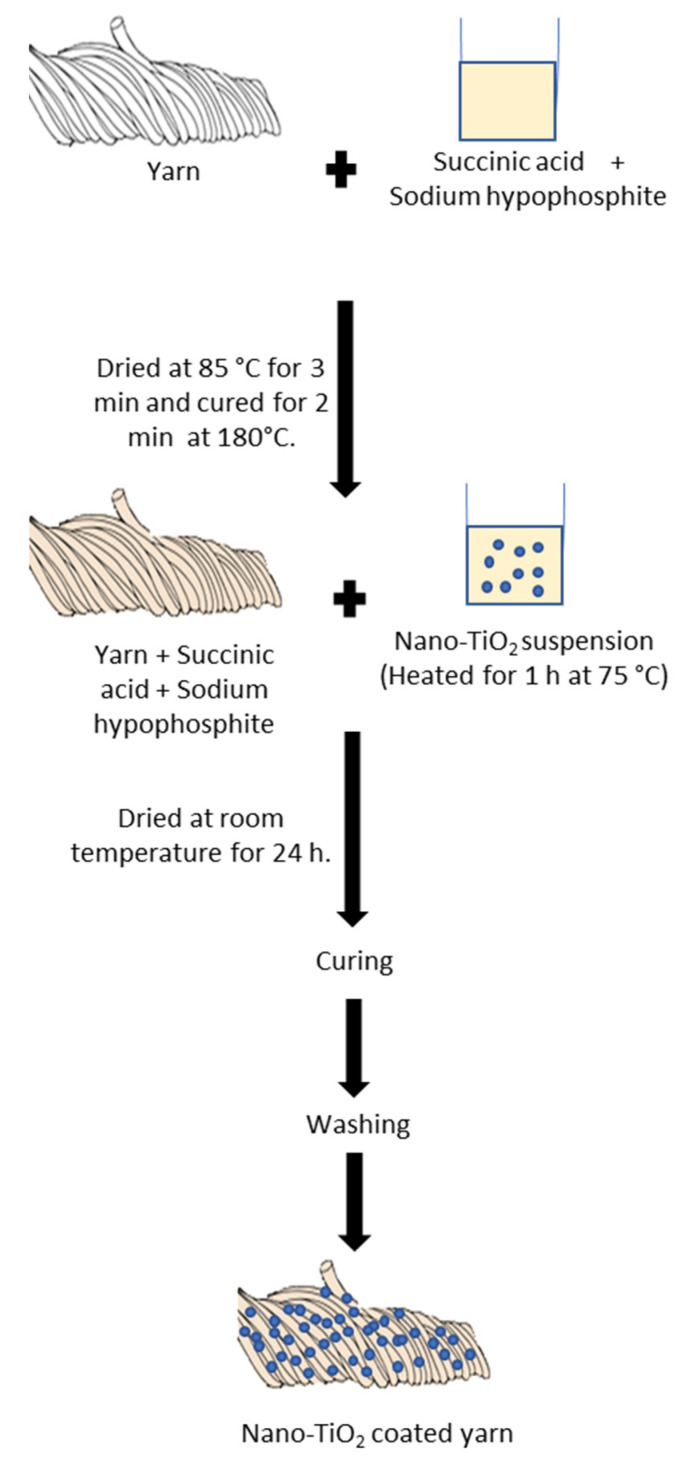
Coating process of the nano-TiO_2_ coated cotton yarn.

This experimental method was adopted and modified from Karimi’s [27] study. The illustration of the coating process is presented in Figure 2. 1 g of nano-TiO_2_ suspension was prepared in 200 mL of distilled water and aged overnight (19–24 h) at room temperature by using an orbital shaker. For the coating process, the cotton yarn was then immersed in 6% of aqueous solution of succinic acid and 4% of sodium hypophosphite (NaH_2_PO_2_) as a catalyst for 1 h at room temperature. The yarn was dried in an oven at 85 °C for 3 min and a curing for 2 min at 180 °C. The cotton yarn loaded with compound succinic acid was immersed into the aqueous suspension of nano-TiO_2_ and was heated for 1 h at 75 °C in a water bath sonicator. The nano-TiO_2_ coated cotton yarn samples were dried at room temperature for 24 h. Then, the sample went through the curing process, followed by washing it with distilled water for 5 min using a water bath sonification at room temperature to eliminate any unfixed nano-titania particles on the surface of the cotton yarn.

### 2.3. Washing Cycles of Nano-TiO_2_ Coated Cotton Yarn

The washing cycles of the nano-TiO_2_ coated cotton yarn were conducted based on a modified AATCC test method 61. All coated cotton yarn samples were washed by the exhaustion dyeing machine. 200 mL of distilled water and 1 g of industrial soap were placed in the exhaustion dyeing machine and rotated at 38 ± 3 °C with a speed of 40 rpm. The nano-TiO_2_ coated yarn samples were washed up to 20 times and divided into 4 phases of washing, which were the 5^th^ wash, 10^th^ wash, 15th wash, and 20^th^ wash. The samples were then tested for morphological structures, yarn weight loss, and self-cleaning. For the yarn weight loss, all the coated cotton yarn samples were weighed before and after each wash cycle.

### 2.4. Scanning Electron Microscopy (SEM), Energy-Dispersive X-ray (EDX) and Ultraviolet–Visible (UV-Vis) Spectroscopy Analysis

A SEM with an attached EDX (Hitachi TM3030, Hitachi High-Technologies Corporation, Japan) was used to characterize the morphological structures and elemental information of the nano-TiO_2_ coated yarn for each washing cycle. Before the analysis, the coated cotton yarn was sputtered with a thin layer of gold to avoid electrostatic charging during testing and to capture a good image of the nano-TiO_2_ coated cotton yarn. The samples were tested using two different magnifications, which were 500× and 5000× magnification, respectively. Meanwhile, the morphological structures and band gap of nano-titania were characterized using SEM (Hitachi TM3030, Hitachi High-Technologies Corporation, Tokyo, Japan) and a UV-Vis spectroscopy (Model Cary 5000, Agilent, CA, USA), respectively.

### 2.5. Self-Cleaning Test by Accelerated Weathering Tester (AWT)

The self-cleaning of the coated cotton yarn was investigated by exposing the nano-TiO_2_ coated yarn samples that had been washed at the 5^th^, 10^th^, 15^th^, and 20^th^ wash cycles under the UV light (315–400 nm) by using an Accelerated Weathering Tester (AWT). All the washed nano-TiO_2_ coated yarn samples were wounded on a heat-resistant board, then 1 mL of coffee was dropped at a consistent height, which was 10 cm from the sample surface. All the samples were mounted on the frame and exposed to the UVA light for 8 h. The evaluation of the self-cleaning of the nano-TiO_2_ coated cotton yarn was conducted through a Hunter Labscan XE spectrophotometer. This machine was used to determine the depth of colour stain of the coated cotton yarn samples, which had been stained with coffee and exposed to the UVA light for 8 h. The depth of the colour stain from the coffee was presented as K/S value, where K and S are corresponded to the absorption and scattering coefficients of the colour stain fabric, respectively. All the samples were compared to the unstained cotton yarn and stained cotton yarn.

## 3. Results and Discussion

### 3.1. Morphological Structures and Band Gap of Nano-TiO_2_ Particles

**Figure 3:**
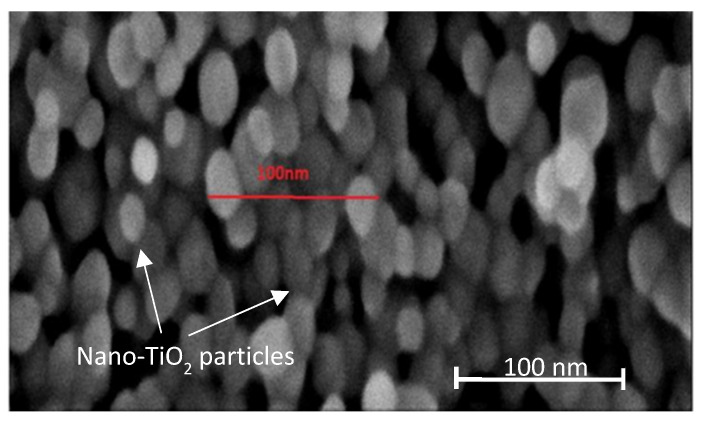
Morphology of nano-TiO_2_ particles. Figure is adapted and modified from [22].

**Figure 4:**
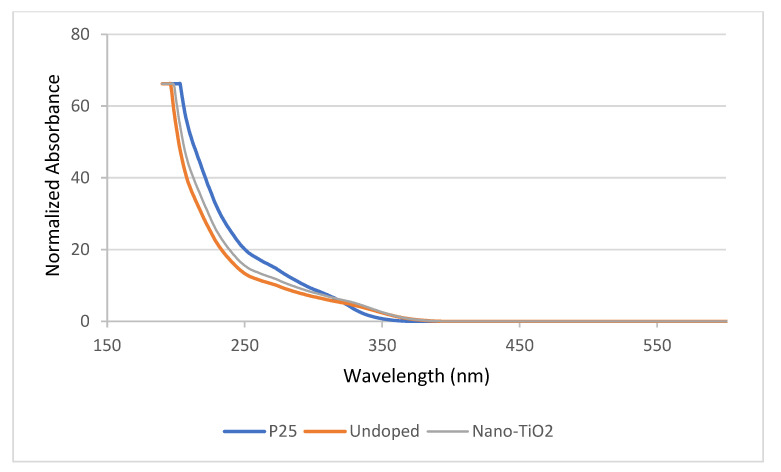
UV-Vis diffuse reflectance spectra of commercial TiO_2_ (P25), undoped TiO_2_, and nano-TiO_2_ samples.

Figure 3 shows the nano-TiO_2_ particles as observed under SEM with the circles, shape, and size within a 20–50 nm range. Details of the morphological structure of the nano-TiO_2_ can be found elsewhere [24]. From our results, the wavelength in the absorption edge for the spectra of the commercial TiO_2_ (P25), undoped TiO_2_, and nano-TiO_2_ were 310 nm, 280 nm, and 290 nm, respectively (Figure 4). It is notable that our synthesized nano-TiO_2_ shifted to a higher wavelength in the absorption edge compared to the undoped TiO_2_ sample.

**Figure 5:**
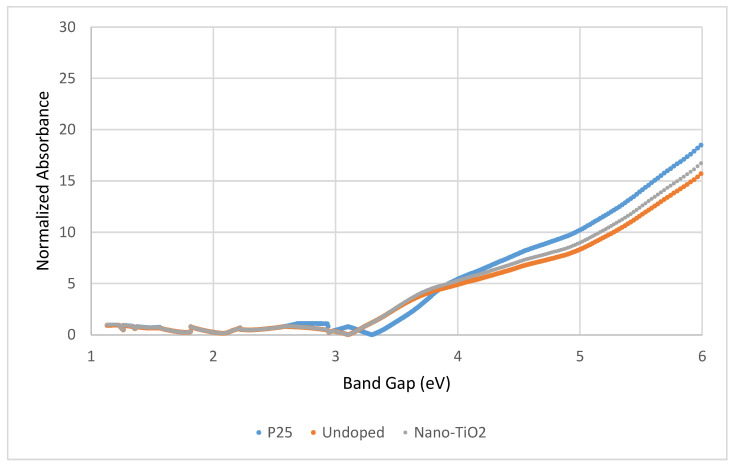
Kubelka-Munk function plots of commercial TiO_2_ (P25), undoped, and nano-TiO_2_.

Meanwhile, Figure 5 shows a Kubelka-Munk function plot for the commercial TiO_2_ (P25), undoped TiO_2_, and nano-TiO_2_ particles. The plot was used to determine the optical bandgap for all samples. Figure 5 illustrates the approximated band gaps of the P25, undoped TiO_2_, and nano-TiO_2_, which are 3.4 eV, 3.06 eV, and 3.06 eV, respectively. In addition, these results are much lower than the TiO_2_ band gap as reported by [28] with 3.23 eV.

The wavelength (nm) in the absorption edge (Figure 5) can be used to indicate the elemental band gap absorption of TiO_2_ resulting from the electron transition from the VB to the CB [29,30]. The higher the wavelength (nm) in the absorption edge, the lower the elemental band gap absorption, which leads to a better photoactivity performance. This proves that the electrons at the VB do not need to absorb high energy from the UV light to excite and escape to the CB. Based on Figure 4 and Figure 5, it can be concluded that the nano-TiO_2_ and undoped samples have a better band gap size compared to the commercial P25. A lower band gap leads to a better absorption edge and better electron transitions from the VB to the CB for enhanced photoactivity performance. With all these properties, the nano-TiO_2_ were then tested for the yarn coating preparations. At the end of the study, cotton with a better adherence of nano-TiO_2_ was obtained and is useful for its self-cleaning property.

### 3.2. The Effect of Washing Cycles of Nano-TiO_2_ Coated Cotton Yarn on Morphological Structure, Elemental Analysis, and Yarn Weight Loss

**Figure 6:**
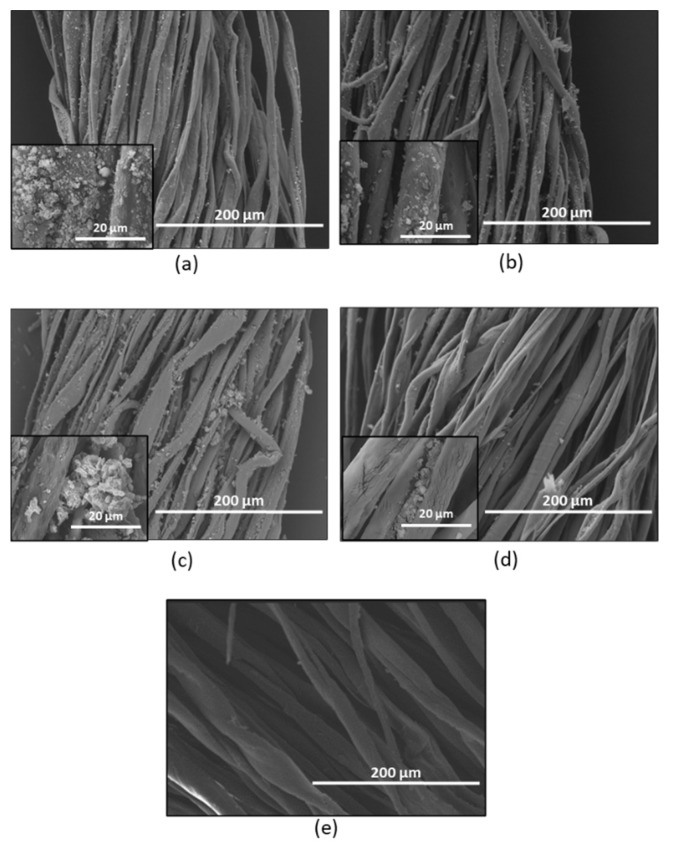
SEM images of nano-TiO_2_ coated cotton yarn at different washing cycles. (**a**) 5^th^ washing cycle, (**b**) 10^th^ washing cycle, (**c**) 15th washing cycle, (**d**) 20^th^ washing cycle, and (**e**) uncoated cotton.

Figure 6a–d depict the morphological analysis of the nano-TiO_2_ coated samples after washing at 500 and 5000 magnification levels. These results show the changes in the morphological structure for all samples at 4 phases of washing cycles that included the 5^th^, 10^th^, 15^th^, and 20^th^, respectively. Meanwhile, Figure 6e presents the image of uncoated cotton yarn, of which apparently the yarn surface was clear, without any presence of the nano-TiO_2_ particles on the surface.

In Figure 6a, the nano-TiO_2_ -coated cotton yarn was spotted numerously on the cotton yarn after the 5^th^ washing cycle. The size of the nano-TiO_2_ particles was in the range of 20 nm to 50 nm. The distribution also seemed to fully cover the surface of cotton yarn at 5000 magnifications. Figure 6b–d depict a reduction of the TiO_2_ nanoparticles after the 10^th^, 15^th^, and 20^th^ washing cycles. The nano-TiO_2_ particles were only seen between the cotton fibres. In addition, an agglomeration of clump was detected at 15^th^ and 20^th^ washing cycles [Figure 6d] and the size of the clump was noticeably larger compared to other washing cycle phases. During washing, the nano-TiO_2_ particles and the succinic acid were expected to agglomerate and form clumps on the cotton yarn surfaces. As reported in several studies, the presence of binders may cause the agglomeration of TiO_2_ particles on the cotton surfaces [31].

**Figure 7:**
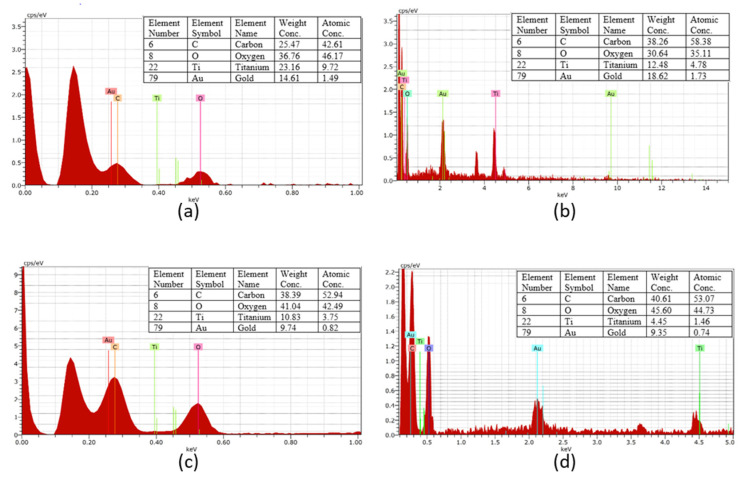
EDX patterns of nano-TiO_2_ coated cotton yarn at (**a**) 5^th^ wash, (**b**) 10^th^ wash, (**c**) 15^th^ wash, and (**d**) 20^th^ wash, respectively. Au indicates the presence of gold sputter coating on the yarn.

The effect of washing cycles on the nano-TiO_2_ coated cotton yarn was further investigated through the EDX analysis [Figure 7a–d]. The existence of the Ti element was observed up to the 20^th^ washing cycle, showing the ability of succinic acid to adhere TiO_2_ nanoparticles on the cotton surfaces. In addition, the weight (%) of Ti was observed to decrease from the 5^th^ to the 20^th^ washing cycle, indicating that some of the nano-TiO_2_ particles had been removed after washing. At the 5^th^ washing cycle, the weight (%) of Ti was approximately 23.16% and was declining to 12.48% at the 10^th^ washing cycle. Then, the weight (%) of the Ti element continued to decline at the 15^th^ washing cycle with approximately 10.83%. Following the 20^th^ washing cycle, the Ti element was further decreased down to 4.45%. From this analysis, it shows the reduction of the Ti element from the coated yarn samples.

To investigate the effectiveness of succinic acid to adhere nano-TiO_2_ particles on cotton yarn after washing, the total weight loss for nano-TiO_2_ coated cotton yarn with succinic acid and nano-TiO_2_ coated cotton yarn without succinic acid was analysed and presented in Table 1. The total weight loss of the yarn sample with succinic acid from the 5^th^ to 20^th^ wash cycles was approximately 10%. Without succinic acid, the total weight loss was higher by approximately 13%. This indicates that as the washing cycles increased from 5 to 20, the nano-TiO_2_ particles leached out rapidly from the cotton yarn surfaces. Only some of the TiO_2_ nanoparticles were left on the cotton yarn.

### 3.3. The Effect of Washing Cycles on Self-Cleaning Properties

**Figure 8:**
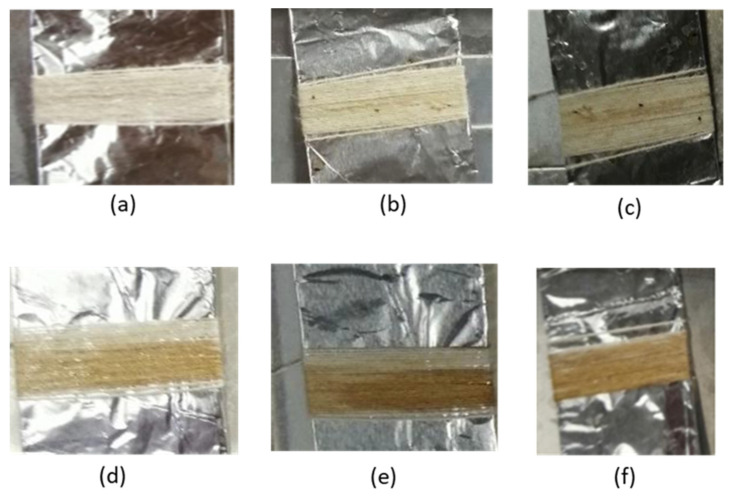
Degradation of coffee stained on nano-TiO_2_ coated cotton yarn after 8 h exposed to UV light. (**a**) Cotton without coffee stain, (**b**) nano-TiO_2_ coated cotton yarn after 5^th^ wash, (**c**) nano-TiO_2_ coated cotton yarn after 10^th^ wash, (**d**) nano-TiO_2_ coated cotton yarn after 15^th^ wash, and (**e**) nano-TiO_2_ coated cotton yarn after 20^th^ wash, and (**f**) cotton with coffee stain, respectively.

**Figure 9:**
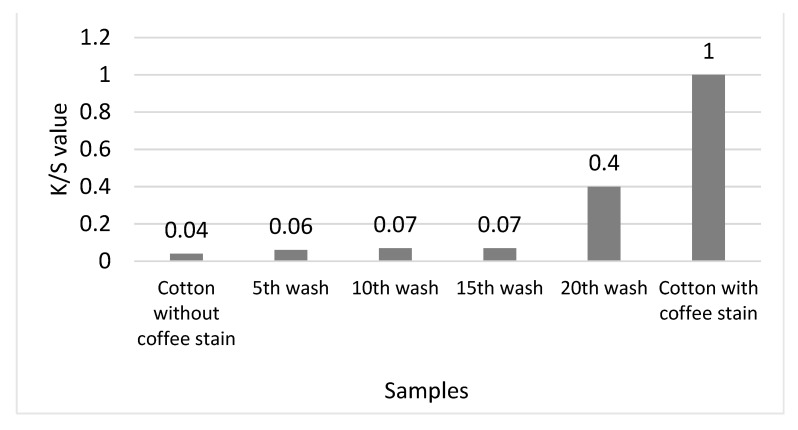
The effect of washing cycles on the degradation of coffee stain.

Self-cleaning properties of nano-TiO_2_ particles coated on cotton yarn had been conducted using the accelerated weathering tester (AWT) machine. The purpose of the experiment was to identify the feasibility of the nano-TiO_2_ to self-clean from the cotton yarn surfaces and also to investigate the effectiveness of the nano-TiO_2_ to self-clean after several washes. In the current study, the samples were stained with coffee. The samples were exposed to UV light for 8 h and the image was taken using a camera. The visual discolouration of the stained TiO_2_ coated cotton yarn is shown in Figure 8a–f. To quantify the degradation or discolouration of the coffee stain, the depth of the colour stain was measured using a hunter lab scan XE spectrophotometer. The colour depth was presented as K/S value and the results were compiled as in Figure 9.

Based on the photograph in Figure 8b, the nano-TiO_2_ coated cotton yarn showed discolouration at the 5^th^ wash cycle. It was observed that the coffee stain was practically removed from the sample. Therefore, this result proves that the TiO_2_ nanoparticles still existed on the cotton yarn after the 5^th^ washing cycle, and the photocatalytic activity of the sample was still effective. Due to a lower band gap (approximately 3.06 eV) of our synthesized nano-TiO_2_, it improves the photoactivity performance of the coated yarn. The TiO_2_ was expected to chemically break down the organic compound into carbon dioxide and water when exposed to UV light. The results agree with those of previous authors, who state that having a lower band gap leads to an improvement in photoactivity performance of TiO_2_ [32]. To strengthen the result of visual discolouration, the sample was tested for a K/S value (Figure 9). The K/S value of yarn after the 5^th^ wash cycle was close to the unstained cotton (cotton without coffee stain), which is 0.04.

The visual discolouration of a coffee stain on the nano-TiO_2_ coated cotton yarn after the 10^th^ washing cycle is presented in Figure 8c. The results show that the coffee stain was still degraded from the sample. The presence of TiO_2_ in the cotton yarn provided the self-cleaning activity of the yarn.

At the 15^th^ wash cycle, the coffee stain on the nano-TiO_2_ coated cotton was still not adequately removed. As illustrated in Figure 8d, the coffee colour was decolourized, yet not as bright as the results at the 5^th^ washing cycle. The current study discovered that the photocatalytic activity was still happening despite the amount of nano-TiO_2_ particles on the cotton yarn surface had been decreased. The K/S values of the sample were still lower than that of the standard stained, indicating that the photocatalytic activity of the samples was still active even after the 15^th^ wash cycle.

The results of visual discolouration of coffee stained on the TiO_2_ coated cotton at the 20^th^ wash cycle, are shown in Figure 8e. The coffee stain was noticeable, showing that the photocatalytic activity was not adequately effective due to the removal of nano-TiO_2_ from the yarn surfaces after the 20^th^ washing cycle. These results were further proven with the K/S value as shown in Figure 9. The K/S value was drastically increased to approximately 0.4. These results indicate that at the 20^th^ wash cycle, the photocatalytic activity was ineffective to decolourize the coffee stain due to the amount of TiO_2_ nanoparticles on the cotton yarn being too little to conduct a photocatalytic activity.

## 4. Conclusions

The effectiveness of succinic acid to keep the adherence of nano-TiO_2_ particles on cotton after washing was investigated in the study. The presence of our synthesized nano-TiO_2_ in cotton yarn demonstrated photocatalytic activities under UV light. As a result, the nano-TiO_2_ coated cotton yarn provided self-cleaning properties up to the 15^th^ wash cycle. In addition, the nano-TiO_2_ coated cotton yarn sample exhibited a distribution of nano-TiO_2_ particles on the cotton yarn surface with no clumps at the 5^th^ wash cycle. As the wash cycle increased up to 20, an agglomeration of clumps was detected on the yarn sample. The analysis also shows that our synthesized nano-TiO_2_ particles have the potential to be used for self-cleaning textiles.

## Figures and Tables

**Table 1:** Weight of nano-TiO_2_ coated cotton yarn with succinic acid and nano-TiO_2_ coated cotton yarn without succinic acid for every wash cycle.

Weight of Sample (g)	Washing Cycle			
	5^th^ Wash	10^th^ Wash	15^th^ Wash	20^th^ Wash
Nano-TiO_2_ coated cotton yarn with succinic acid	2.50	2.46	2.36	2.25
Nano-TiO_2_ coated cotton yarn without succinic acid	2.46	2.36	2.26	2.14
